# Supplementary material for: Diet and gut microbiome of skipjack tuna (Katsuwonus pelamis) as indicators of environmental changes
Source: PLoS One. 2026 Apr 27;21(4):e0346882. doi: 10.1371/journal.pone.0346882 (PMC13119836; doi:10.1371/journal.pone.0346882)
Supplement: S1 Table — (DOCX) [file pone.0346882.s003.docx]

# Diet and gut microbiome of skipjack tuna (*Katsuwonus pelamis*) as indicators of environmental changes

Yufei Zhou^1*^, Alejandro Trujillo-González^1^, Simon Nicol^1, 2^, Roger Huerlimann^3^, Stephen D. Sarre^1^, Dianne Gleeson^1^

^1^ Centre for Conservation Ecology and Genomics, EcoDNA group, University of Canberra, 11 Kirinari Street, Canberra, ACT, 2617, Australia

^2^ Oceanic Fisheries Programme, Pacific Community, Noumea, New Caledonia

^3^ Marine Climate Change Unit, Okinawa Institute of Science and Technology Graduate University, Onna-son, Okinawa, Japan

^*^Correspondence: Yufei Zhou, Yufei.zhou@canberra.edu.au

**S1 Table.** Results for Shapiro-Wilk normality test for alpha diversity indices.

|  | Diet | | | Gut microbiome | | | |
| --- | --- | --- | --- | --- | --- | --- | --- |
|  | Shannon diversity | Chao1 richness | Simpsons evenness | Shannon diversity | Chao1 richness | Simpsons evenness | Abundance of core microbiota |
| W | 0.95 | 0.98 | 0.86 | 0.98 | 0.81 | 0.93 | 0.91 |
| *p* | 0.01 | 0.05 | < 0.01 | 0.02 | < 0.01 | < 0.01 | < 0.01 |
