# Supplementary material for: Diet and gut microbiome of skipjack tuna (Katsuwonus pelamis) as indicators of environmental changes
Source: PLoS One. 2026 Apr 27;21(4):e0346882. doi: 10.1371/journal.pone.0346882 (PMC13119836; doi:10.1371/journal.pone.0346882)
Supplement: S2 Table — (DOCX) [file pone.0346882.s004.docx]

# Diet and gut microbiome of skipjack tuna (*Katsuwonus pelamis*) as indicators of environmental changes

Yufei Zhou^1*^, Alejandro Trujillo-González^1^, Simon Nicol^1, 2^, Roger Huerlimann^3^, Stephen D. Sarre^1^, Dianne Gleeson^1^

^1^ Centre for Conservation Ecology and Genomics, EcoDNA group, University of Canberra, 11 Kirinari Street, Canberra, ACT, 2617, Australia

^2^ Oceanic Fisheries Programme, Pacific Community, Noumea, New Caledonia

^3^ Marine Climate Change Unit, Okinawa Institute of Science and Technology Graduate University, Onna-son, Okinawa, Japan

^*^Correspondence: Yufei Zhou, [Yufei.zhou@canberra.edu.au](mailto:Yufei.zhou@canberra.edu.au)

**S2 Table.** Kruskal-Wallis rank sum test for the association between diet diversity of skipjack tuna and categorical explanatory variables.

|  |  | School | Sex | Health | FADs | ENSO (year) |
| --- | --- | --- | --- | --- | --- | --- |
| Shannon  diversity | χ^2^ | 13.7 | 0.8 | 4.7 | 0.01 | 0.5 |
|  | Df | 13 | 2 | 2 | 1 | 1 |
|  | *p* | 0.32 | 0.66 | 0.10 | 0.92 | 0.47 |
| ChaoI richness | χ^2^ | 10.5 | 0.01 | 4.4 | 0.02 | 0.6 |
|  | Df | 13 | 2 | 2 | 1 | 1 |
|  | *p* | 0.57 | 0.99 | 0.11 | 0.89 | 0.44 |
| Simpsons evenness | χ^2^ | 7.4 | 1.6 | 2.2 | 0.03 | 0.16 |
|  | Df | 13 | 2 | 2 | 1 | 1 |
|  | *p* | 0.83 | 0.45 | 0.33 | 0.86 | 0.69 |
