# Supplementary material for: Diet and gut microbiome of skipjack tuna (Katsuwonus pelamis) as indicators of environmental changes
Source: PLoS One. 2026 Apr 27;21(4):e0346882. doi: 10.1371/journal.pone.0346882 (PMC13119836; doi:10.1371/journal.pone.0346882)
Supplement: S3 Table — (DOCX) [file pone.0346882.s005.docx]

# Diet and gut microbiome of skipjack tuna (*Katsuwonus pelamis*) as indicators of environmental changes

Yufei Zhou^1*^, Alejandro Trujillo-González^1^, Simon Nicol^1, 2^, Roger Huerlimann^3^, Stephen D. Sarre^1^, Dianne Gleeson^1^

^1^ Centre for Conservation Ecology and Genomics, EcoDNA group, University of Canberra, 11 Kirinari Street, Canberra, ACT, 2617, Australia

^2^ Oceanic Fisheries Programme, Pacific Community, Noumea, New Caledonia

^3^ Marine Climate Change Unit, Okinawa Institute of Science and Technology Graduate University, Onna-son, Okinawa, Japan

^*^Correspondence: Yufei Zhou, [Yufei.zhou@canberra.edu.au](mailto:Yufei.zhou@canberra.edu.au)

**S3 Table.** GAM test for the association between diet diversity of skipjack tuna and continuous explanatory variables

|  |  | Length | Chlorophyll | SST | SOI |
| --- | --- | --- | --- | --- | --- |
| Shannon  diversity | Estimate std | 0.006 | 0.58 | 0.13 | 0.002 |
|  | error | 0.006 | 0.51 | 0.14 | 0.005 |
|  | t | 1.02 | 1.14 | 0.90 | 0.40 |
|  | *p* | 0.31 | 0.26 | 0.37 | 0.69 |
| ChaoI richness | Estimate std | 0.01 | 0.33 | 0.24 | 0.002 |
|  | error | 0.02 | 1.44 | 0.40 | 0.01 |
|  | t | 0.60 | 0.23 | 0.60 | 0.17 |
|  | *p* | 0.56 | 0.82 | 0.56 | 0.87 |
| Simpsons evenness | Estimate std | -0.002 | 0.18 | -0.05 | -0.002 |
|  | error | < 0.001 | 0.32 | 0.09 | < 0.001 |
|  | t | -0.55 | 0.57 | -0.56 | -0.58 |
|  | *p* | 0.59 | 0.57 | 0.58 | 0.57 |
