# Supplementary material for: Diet and gut microbiome of skipjack tuna (Katsuwonus pelamis) as indicators of environmental changes
Source: PLoS One. 2026 Apr 27;21(4):e0346882. doi: 10.1371/journal.pone.0346882 (PMC13119836; doi:10.1371/journal.pone.0346882)
Supplement: S4 Table — (DOCX) [file pone.0346882.s006.docx]

# Diet and gut microbiome of skipjack tuna (*Katsuwonus pelamis*) as indicators of environmental changes

Yufei Zhou^1*^, Alejandro Trujillo-González^1^, Simon Nicol^1, 2^, Roger Huerlimann^3^, Stephen D. Sarre^1^, Dianne Gleeson^1^

^1^ Centre for Conservation Ecology and Genomics, EcoDNA group, University of Canberra, 11 Kirinari Street, Canberra, ACT, 2617, Australia

^2^ Oceanic Fisheries Programme, Pacific Community, Noumea, New Caledonia

^3^ Marine Climate Change Unit, Okinawa Institute of Science and Technology Graduate University, Onna-son, Okinawa, Japan

^*^Correspondence: Yufei Zhou, [Yufei.zhou@canberra.edu.au](mailto:Yufei.zhou@canberra.edu.au)

**S4 Table.** Kruskal-Wallis rank sum test for the association between gut microbiome diversity of skipjack tuna and categorical explanatory variables.

|  |  | School | Sex | Length | Health | FADs | Diet cat | ENSO (year) |
| --- | --- | --- | --- | --- | --- | --- | --- | --- |
| Shannon diversity | χ^2^ | 20.1 | 1.1 | 15.0 | 6.3 | 0.1 | 2.3 | 2.2 |
|  | Df | 14 | 2 | 19 | 2 | 1 | 3 | 1 |
|  | *p* | 0.13 | 0.59 | 0.66 | 0.04 | 0.79 | 0.51 | 0.13 |
| ChaoI richness | χ^2^ | 33.7 | 0.5 | 25.4 | 1.9 | 1.5 | 2.6 | 0.4 |
|  | Df | 14 | 2 | 19 | 2 | 1 | 3 | 1 |
|  | *p* | **0.002** | 0.76 | 0.11 | 0.39 | 0.23 | 0.46 | 0.52 |
| Simpsons evenness | χ^2^ | 26.1 | 0.3 | 13.0 | 6.2 | 1.8 | 0.2 | 4.9 |
|  | Df | 14 | 2 | 19 | 2 | 1 | 3 | 1 |
|  | *p* | **0.03** | 0.84 | 0.79 | 0.44 | 0.18 | 0.98 | **0.03** |
| Abundance of core microbiota | χ^2^ | 32.5 | 2.7 | 26.6 | 1.8 | 0.3 | 3.1 | 2.6 |
|  | Df | 14 | 2 | 19 | 2 | 1 | 3 | 1 |
|  | *p* | **0.003** | 0.26 | 0.09 | 0.41 | 0.60 | 0.37 | 0.11 |
