# Supplementary material for: Diet and gut microbiome of skipjack tuna (Katsuwonus pelamis) as indicators of environmental changes
Source: PLoS One. 2026 Apr 27;21(4):e0346882. doi: 10.1371/journal.pone.0346882 (PMC13119836; doi:10.1371/journal.pone.0346882)
Supplement: S5 Table — (DOCX) [file pone.0346882.s007.docx]

# Diet and gut microbiome of skipjack tuna (*Katsuwonus pelamis*) as indicators of environmental changes

Yufei Zhou^1*^, Alejandro Trujillo-González^1^, Simon Nicol^1, 2^, Roger Huerlimann^3^, Stephen D. Sarre^1^, Dianne Gleeson^1^

^1^ Centre for Conservation Ecology and Genomics, EcoDNA group, University of Canberra, 11 Kirinari Street, Canberra, ACT, 2617, Australia

^2^ Oceanic Fisheries Programme, Pacific Community, Noumea, New Caledonia

^3^ Marine Climate Change Unit, Okinawa Institute of Science and Technology Graduate University, Onna-son, Okinawa, Japan

^*^Correspondence: Yufei Zhou, [Yufei.zhou@canberra.edu.au](mailto:Yufei.zhou@canberra.edu.au)

**S5 Table.** GAM test for the association between gut microbiome diversity of skipjack tuna and continuous explanatory variables

|  | GAM | Length | RA of fish in diet | Chlorophyll | SST | SOI |
| --- | --- | --- | --- | --- | --- | --- |
| Shannon diversity  (Gamma family) | Estimate std | -1.1 | -0.09 | 0.02 | 0.02 | 0.001 |
|  | error | 0.06 | 0.10 | 0.02 | 0.06 | 0.002 |
|  | t | -1.76 | -0.87 | 0.99 | 0.37 | 0.52 |
|  | *p* | 0.08 | 0.39 | 0.32 | 0.71 | 0.60 |
| ChaoI richness  (Gamma family) | Estimate std | -0.04 | -0.002 | -0.0007 | -0.002 | -0.09 |
|  | error | 0.02 | 0.004 | 0.0007 | 0.002 | 0.005 |
|  | t | -1.94 | -0.55 | -1.01 | -1.05 | -1.93 |
|  | *p* | 0.06 | 0.59 | 0.32 | 0.29 | **0.04** |
| Simpsons evenness  (Quasi-binomial family) | Estimate std | 6.69 | 0.30 | 0.59 | 0.24 | 0.01 |
|  | error | 4.10 | 0.91 | 0.42 | 0.16 | 0.005 |
|  | t | 1.62 | 0.33 | 1.40 | 1.50 | 2.32 |
|  | *p* | 0.11 | 0.74 | 0.16 | 0.14 | **0.02** |
| Abundance of core microbiota  (Quasi-binomial family) | Estimate std | -1.21 | 0.39 | -0.09 | -0.11 | -4.64 |
|  | error | 1.42 | 0.23 | 0.05 | 0.13 | 3.51 |
|  | t | -0.86 | 1.73 | -1.76 | -0.83 | -1.32 |
|  | *p* | 0.39 | 0.09 | 0.08 | 0.41 | 0.19 |
