# Supplementary material for: Diet and gut microbiome of skipjack tuna (Katsuwonus pelamis) as indicators of environmental changes
Source: PLoS One. 2026 Apr 27;21(4):e0346882. doi: 10.1371/journal.pone.0346882 (PMC13119836; doi:10.1371/journal.pone.0346882)
Supplement: S6 Table — (DOCX) [file pone.0346882.s008.docx]

# Diet and gut microbiome of skipjack tuna (*Katsuwonus pelamis*) as indicators of environmental changes

Yufei Zhou^1*^, Alejandro Trujillo-González^1^, Simon Nicol^1, 2^, Roger Huerlimann^3^, Stephen D. Sarre^1^, Dianne Gleeson^1^

^1^ Centre for Conservation Ecology and Genomics, EcoDNA group, University of Canberra, 11 Kirinari Street, Canberra, ACT, 2617, Australia

^2^ Oceanic Fisheries Programme, Pacific Community, Noumea, New Caledonia

^3^ Marine Climate Change Unit, Okinawa Institute of Science and Technology Graduate University, Onna-son, Okinawa, Japan

^*^Correspondence: Yufei Zhou, [Yufei.zhou@canberra.edu.au](mailto:Yufei.zhou@canberra.edu.au)

**S6 Table.** PERMANOVA result for beta diversity of gut microbiome of skipjack tuna in association with explanatory variables.

| Total sum scaling (TSS) transformed data and unique fraction (UniFrac) distance metric | | | | | | | | | |
| --- | --- | --- | --- | --- | --- | --- | --- | --- | --- |
| PERMDIST test | School | Sex | Length | Health | FADs | ENSO (year) | Chlorophyll | SST | SOI |
| Df | 14 | 2 | 18 | 2 | 1 | 1 | 8 | 1- | 14 |
| Sum of Squares | 0.03 | 0.27 | 1.02 | 0.03 | 0.01 | 0.003 | 0.07 | 0.06 | 0.02 |
| Mean of Squares | 0.002 | 0.14 | 0.06 | 0.01 | 0.01 | 0.003 | 0.01 | 0.01 | 0.001 |
| F | 0.65 | 75.3 | 22.3 | 7.79 | 4.15 | 1.40 | 2.70 | 1.89 | 0.35 |
| Pr | 0.83 | **0.001** | **0.001** | **0.004** | **0.04** | 0.26 | **0.01** | 0.06 | 0.98 |
| PERMANOVA test | School | Sex | Length | Health | FADs | ENSO (year) | Chlorophyll | SST | SOI |
| Df | 14 | 2 | 1 | 2 | 1 | 1 | 1 | 1 | 1 |
| Sum of Squares | 6.07 | 0.34 | 0.21 | 0.62 | 0.62 | 0.83 | 0.77 | 0.20 | 0.80 |
| R^2^ | 0.16 | 0.01 | 0.01 | 0.02 | 0.02 | 0.02 | 0.02 | 0.01 | 0.02 |
| F | 1.67 | 0.74 | 0.92 | 1.39 | 2.77 | 3.03 | 2.81 | 0.90 | 2.93 |
| *p* | **0.001** | 0.79 | 0.50 | 0.12 | **0.01** | **0.001** | **0.001** | 0.49 | **0.001** |

| Centered Log-Ratio (CLR) transformed data and Euclidean distance metric | | | | | | | | | |
| --- | --- | --- | --- | --- | --- | --- | --- | --- | --- |
| PERMDIST test | School | Sex | Length | Health | FADs | ENSO (year) | Chlorophyll | SST | SOI |
| Df | 14 | 2 | 18 | 2 | 1 | 1 | 8 | 10 | 14 |
| Sum of Squares | 287.2 | 450.5 | 1816 | 8.71 | 44.3 | 0.3 | 286.8 | 151.8 | 282.1 |
| Mean of Squares | 20.1 | 225.3 | 100.9 | 4.4 | 44.3 | 0.3 | 35.8 | 15.2 | 21.7 |
| F | 1.17 | 12.5 | 5.9 | 0.2 | 2.4 | 0.01 | 2.1 | 0.9 | 1.3 |
| Pr | 0.31 | **0.002** | **0.001** | 0.78 | 0.14 | 0.91 | **0.04** | 0.59 | 0.24 |
| PERMANOVA test | School | Sex | Length | Health | FADs | ENSO (year) | Chlorophyll | SST | SOI |
| Df | 14 | 2 | 1 | 2 | 1 | 1 | 1 | 1 | 1 |
| Sum of Squares | 9745 | 833 | 678 | 1274 | 1429 | 1435 | 1360 | 578 | 1396 |
| R^2^ | 0.16 | 0.13 | 0.01 | 0.02 | 0.02 | 0.02 | 0.02 | 0.01 | 0.02 |
| F | 1.61 | 0.91 | 1.48 | 1.39 | 3.16 | 3.18 | 3.01 | 1.26 | 3.10 |
| *p* | **0.001** | 0.60 | 0.06 | 0.21 | **0.001** | **0.001** | **0.001** | 0.09 | **0.001** |
