# Supplementary material for: Diet and gut microbiome of skipjack tuna (Katsuwonus pelamis) as indicators of environmental changes
Source: PLoS One. 2026 Apr 27;21(4):e0346882. doi: 10.1371/journal.pone.0346882 (PMC13119836; doi:10.1371/journal.pone.0346882)

# Diet and gut microbiome of skipjack tuna (*Katsuwonus pelamis*) as indicators of environmental changes

Yufei Zhou^1*^, Alejandro Trujillo-González^1^, Simon Nicol^1, 2^, Roger Huerlimann^3^, Stephen D. Sarre^1^, Dianne Gleeson^1^

^1^ Centre for Conservation Ecology and Genomics, EcoDNA group, University of Canberra, 11 Kirinari Street, Canberra, ACT, 2617, Australia

^2^ Oceanic Fisheries Programme, Pacific Community, Noumea, New Caledonia

^3^ Marine Climate Change Unit, Okinawa Institute of Science and Technology Graduate University, Onna-son, Okinawa, Japan

^*^Correspondence: Yufei Zhou, Yufei.zhou@canberra.edu.au

**S3 Fig. Partial dependence plots showing relationships between bacterial family abundances and predicted SOI values.** Each panel shows the marginal effect of one bacterial family's log10-transformed abundance (x-axis) on predicted SOI (y-axis) while averaging over the effects of other families in the random forest model.


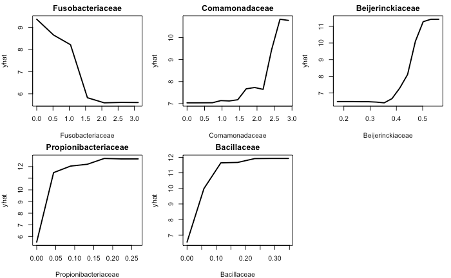

Supplement: S3 Fig — Each panel shows the marginal effect of one bacterial family’s log10-transformed abundance (x-axis) on predicted SOI (y-axis) while averaging over the effects of other families in the random forest model. (DOCX) [file pone.0346882.s010.docx]
