# Supplementary material for: Diet and gut microbiome of skipjack tuna (Katsuwonus pelamis) as indicators of environmental changes
Source: PLoS One. 2026 Apr 27;21(4):e0346882. doi: 10.1371/journal.pone.0346882 (PMC13119836; doi:10.1371/journal.pone.0346882)
Supplement: S7 Table — Abundances of five important families (Fusobacteriaceae, Bacillaceae, Propionibacteriaceae, Beijerinckiaceae, and Comamonadaceae) are explanatory variables, and ENSO event is the prediction variable. (DOCX) [file pone.0346882.s011.docx]

# Diet and gut microbiome of skipjack tuna (*Katsuwonus pelamis*) as indicators of environmental changes

Yufei Zhou^1*^, Alejandro Trujillo-González^1^, Simon Nicol^1, 2^, Roger Huerlimann^3^, Stephen D. Sarre^1^, Dianne Gleeson^1^

^1^ Centre for Conservation Ecology and Genomics, EcoDNA group, University of Canberra, 11 Kirinari Street, Canberra, ACT, 2617, Australia

^2^ Oceanic Fisheries Programme, Pacific Community, Noumea, New Caledonia

^3^ Marine Climate Change Unit, Okinawa Institute of Science and Technology Graduate University, Onna-son, Okinawa, Japan

^*^Correspondence: Yufei Zhou, [Yufei.zhou@canberra.edu.au](mailto:Yufei.zhou@canberra.edu.au)

**S7 Table.** **Leave one school out internal test result for random forest model (RFM).** Abundances of five important families (Fusobacteriaceae, Bacillaceae, Propionibacteriaceae, Beijerinckiaceae, and Comamonadaceae) are explanatory variables, and ENSO event is the prediction variable.

| Predicted  Actual | La Niña | El Niño |
| --- | --- | --- |
| La Niña | 7 | 0 |
| El Niño | 0 | 8 |
