# Supplementary material for: Diet and gut microbiome of skipjack tuna (Katsuwonus pelamis) as indicators of environmental changes
Source: PLoS One. 2026 Apr 27;21(4):e0346882. doi: 10.1371/journal.pone.0346882 (PMC13119836; doi:10.1371/journal.pone.0346882)
Supplement: Attachment — Submitted filename: PlosOne_rebuttal_letter.docx [file pone.0346882.s013.docx]

# Journal Requirements:

1. Please ensure that your manuscript meets PLOS ONE's style requirements, including those for file naming. The PLOS ONE style templates can be found at https://journals.plos.org/plosone/s/file?id=wjVg/PLOSOne_formatting_sample_main_body.pdf and https://journals.plos.org/plosone/s/file?id=ba62/PLOSOne_formatting_sample_title_authors_affiliations.pdf

**Author response:**

We have reviewed and updated the manuscript to ensure compliance with PLOS ONE's style requirements, including proper file naming conventions, reference formatting, and figure/table formatting guidelines.

2. In your Methods section, please provide additional information regarding the permits you obtained for the work. Please ensure you have included the full name of the authority that approved the field site access and, if no permits were required, a brief statement explaining why.

**Author response:**

We have added a complete ethics statement to the Methods section. As confirmed by our co-authors at the Pacific Community, no field access permits were required for this study, as all samples originated from commercial fishery catches.

3. Thank you for stating the following financial disclosure:

This work was supported by the University of Canberra and by the European Union “Pacific-European-Union-Marine-Partnership” Programme (agreement FED/2018/397-941) grant to the Pacific Community. This publication was produced with the financial support of the European Union and Sweden. Its contents are the sole responsibility of the authors and do not necessarily reflect the views of the European Union and Sweden.

Please state what role the funders took in the study. If the funders had no role, please state: "The funders had no role in study design, data collection and analysis, decision to publish, or preparation of the manuscript."

If this statement is not correct you must amend it as needed.

Please include this amended Role of Funder statement in your cover letter; we will change the online submission form on your behalf.

**Author response:**

We have included this statement in both cover letter and in the Funding sources section in the manuscript.

4. Please include your full ethics statement in the ‘Methods’ section of your manuscript file. In your statement, please include the full name of the IRB or ethics committee who approved or waived your study, as well as whether or not you obtained informed written or verbal consent. If consent was waived for your study, please include this information in your statement as well.

**Author response:**

We have added a complete ethics statement to the Methods section. The statement specifies that gut lining samples were collected from commercially caught skipjack tuna during routine operations, with no live animals handled for experimental purposes. All sampling followed standard commercial fishing practices and regional fisheries management protocols.

5. Please remove all personal information, ensure that the data shared are in accordance with participant consent, and re-upload a fully anonymized data set.

Note: spreadsheet columns with personal information must be removed and not hidden as all hidden columns will appear in the published file.

Additional guidance on preparing raw data for publication can be found in our Data Policy (https://journals.plos.org/plosone/s/data-availability#loc-human-research-participant-data-and-other-sensitive-data) and in the following article: http://www.bmj.com/content/340/bmj.c181.long.

**Author response:**

We have reviewed all data files submitted with this manuscript to ensure no personal information is included. This study involves marine fish samples collected from commercial fisheries operations and does not involve human participants. We confirm that: (1) All spreadsheet files contain only scientific data (sample identifiers, fish measurements, date and locations, and environmental variables) with no personal information in any columns. (2) No columns are hidden in any data files. (3) File metadata (author, last modified by) has been cleared from all supplementary files. (4) The only names appearing in the manuscript are standard author citations in the reference list and necessary attributions for external datasets (GMB and TGM projects), which are required for scientific transparency and reproducibility.

6. If the reviewer comments include a recommendation to cite specific previously published works, please review and evaluate these publications to determine whether they are relevant and should be cited. There is no requirement to cite these works unless the editor has indicated otherwise.

**Author response:**

The reviewers did not recommend any specific citations to be added to the manuscript. All citations in the revised manuscript were selected based on their relevance to our study design, methodology, results, and interpretation.

# Reviewer #1:

Title and Abstract

Strengths: Clear, accurate, and aligned with results; avoids overstating novelty. The abstract correctly contrasts weak diet signals with stronger microbiome responses, and it names the five key families and the modeling approach.

Suggestions:

1. Tone down “successfully predicted SOI” and add a brief caveat that external validation was mixed (good on TGM, poor on GMB), likely due to range mismatch and storage effects.

2. Consider noting the short, two-time-point design to set expectations.

**Author response:**

Thank you for recognizing the clarity and accuracy of our abstract and valuable suggestions. We have revised the abstract to address both suggestions.

**Revised section:**

Lines 32 – 37: A random forest model showed potential for ENSO phase classification based on the abundances of these five families, achieve high accuracy in internal validation, though the performance of external validation was mixed due to database differences . This study highlights the potential of skipjack tuna gut microbiome as indicators of rapid environmental changes, while acknowledging that the short sampling period requires longer-term validation across multiple ENSO cycles.

Introduction

Strengths: The motivation for using predator guts (diet + microbiome) as sentinel readouts is well framed; the ENSO context is clear; the gap (microbiome responses in skipjack) is articulated.

Suggestions: When attributing functional roles to higher-taxonomic ranks (e.g., family-level “probiotic” or immune associations), emphasize that function cannot be inferred directly from amplicon data and that families are ecologically broad. (You can preserve these links but frame them as hypotheses, supported by cited literature.)

**Author response:**

We have revised the Introduction to use more cautious language about gut microbiome functions, changing "is closely related to" to "has been associated with" when discussing relationships between microbiome and host physiology. Since the Introduction provides general context rather than discussing specific bacterial families, we reserved detailed discussion of family-level taxonomic limitations for the Discussion section where we interpret our microbiome results. Please refer to our response to Comment 2 in Discussion and Conclusions Section.

**Revised section:**

Lines 62 – 63: Second, the gut microbiome, which has been associated with the host’s nutritional provisioning, metabolism, and immune system functionality [20–25] …

Materials and Methods

Study design and sampling

Strengths: School-based design spanning a strong phase transition is compelling; within-school replication (∼10 fish) is practical.

Major points to address:

1. Temporal/Spatial confounding: All La Niña schools were sampled in December and all El Niño schools in March; locations span 05–09°S, 158–161°E. Please discuss potential spatial confounding and time-lag effects explicitly (ENSO signals propagate, and ecological responses can lag weeks–months). A map with school positions and dates would help.

**Author response:**

We acknowledge this important limitation and have added explicit discussion of potential confounding effects. However, several lines of evidence support that our observed microbiome patterns reflect direct ENSO effects rather than spatial artifacts: (1) The 4 - 5 degrees latitudinal sampling range is modest relative to skipjack tuna migration patterns and represents regional population variation rather than distinct stocks; (2) The absence of significant diet changes between sampling periods, despite clear microbiome shifts, suggests that gut microbiome responses to environmental change are more rapid and direct than traditional ecological indicators; (3) Among all environmental variables tested (SST, chlorophyll-a, SOI), only SOI significantly correlated with microbiome variation, supporting ENSO-specific rather than general temporal effects.

However, our two-timepoint design cannot definitively separate ENSO effects from seasonal variations. We have revised the manuscript to acknowledge this limitation and added a map showing sampling locations and dates as requested in the supplementary file (S1 Fig).

**Revised section**

Lines 107 – 110: While this design cannot entirely exclude potential confounding effects, the spatial range is modest relative to skipjack tuna migration patterns and represents regional rather than large scale variation [40, 41].

Lines 473 – 478: Spatial confounding might be less significant because the 4 - 5 degree latitudinal sampling range represents modest variation relative to skipjack tuna migration patterns and regional stock distribution [40,41,84]. However, we acknowledge that our two-timepoint design cannot definitively separate ENSO effects from seasonal variations. Systematic sampling across multiple ENSO cycles at fixed locations would be required to conclusively separate ENSO effects from temporal and spatial variation.

2. Independence & hierarchical structure: Analyses sometimes operate at the individual level and sometimes at the school level (e.g., SOI models use school-level log10 abundances). Given the evident school effect in microbiome beta diversity (R² ≈ 0.15), please account for school as a grouping factor (random effect or blocking) wherever appropriate.

**Author response:**

We acknowledge this important statistical consideration. Upon review, we found that school effects were appropriately handled in our analyses: (1) school was included as a factor in PERMANOVA tests, (2) Kruskal-Wallis tests for diversity indices inherently account for school-level grouping, and (3) SOI prediction models appropriately used school-level data.

Regarding the SOI prediction models specifically, we have re-examined our approach in light of this comment. Since SOI varies by sampling date, each school represents a unique SOI value, making school-level analysis the statistically appropriate approach. Our GAM and Random Forest models using school-level aggregated bacterial abundances to predict school-level SOI values are methodologically sound because both predictors and response variables operate at the same hierarchical level. Adding school as a random effect would be inappropriate in this context since SOI is inherently a school-level variable rather than an individual fish characteristic.

**Revised section:**

Lines 278 – 280: Bacterial family abundances were aggregated at the school level by calculating log10-transformed mean abundances, since SOI values correspond to sampling dates and locations that are consistent within each school.

Lines 400 – 401: A GAM and a RFM were constructed and compared, using the school-level log10-transformed abundance of the five families as predictor variables and SOI as the predicted variable.

Laboratory and library prep

Strengths: Clear protocols; blocking primer for host COI; triplicate PCRs; negative controls at every stage; inclusion of ZymoBIOMICS community standards.

Suggestions: State explicitly whether any decontamination algorithm (e.g., decontam) was applied in addition to negative-control screening, especially relevant for families such as Propionibacteriaceae/Cutibacteriaceae that can be skin-associated. (You do note clean negatives.)

**Author response:**

We appreciate this suggestion. We did not apply computational decontamination algorithms (such as decontam) beyond visual inspection of negative controls. Our negative controls showed no detectable DNA concentrations and were excluded from sequencing, indicating minimal contamination during sample processing. We acknowledge that families like Propionibacteriaceae can be skin-associated, but several factors support the validity of our detection: (1) samples were processed immediately after capture with sterile techniques, (2) negative controls from each processing stage showed no amplification, and (3) Propionibacteriaceae abundance patterns correlated specifically with environmental variables rather than showing random distribution that would suggest contamination artifacts.

However, we acknowledge that computational decontamination would strengthen future studies and have noted this in our revised Methods section.

**Revised section:**

Lines 296 – 297: No additional computational decontamination algorithms were applied beyond negative control screening.

Bioinformatics and normalization

Strengths: DADA2, unambiguous phylum-level assignment, chloroplast/mitochondria removal, ANCOM-BC2 for differential abundance, and UniFrac for beta diversity—all standards.

Major points to address:

1. Alpha diversity computed on non-normalized counts: The text says alpha diversity indices were computed on non-normalized data. This can confound diversity with sequencing depth. Please either rarefy, include depth as a covariate, or use estimators robust to depth (e.g., breakaway/DivNet).

**Author response:**

We appreciate this suggestion. We have revised our alpha diversity analysis by rarefying all samples to equal sequencing depth before calculating diversity indices. The rarefied analysis shows identical statistical significance patterns to our original analysis, confirming that our findings were not confounded by sequencing depth variation. We have updated our Methods section to describe the rarefaction procedure and revised Figure 3A and Supplementary File 2 accordingly.

**Revised section:**

Lines 223 – 226: Prior to alpha diversity analysis, samples were rarefied to equal sequencing depth (5019 reads per sample for diet data and 4017 reads per sample for microbiome data) using the phyloseq package (version 1.38.0) [51]. Shannon diversity [57], Chao1 species richness index [58], Simpson’s species evenness index [59] were calculated for rarefied diet and gut microbiome data using the microbiome package (version 1.23.1) [54].

2. PERMANOVA assumptions: Before interpreting PERMANOVA, test multivariate dispersion (PERMDISP/betadisper). Unequal dispersion across groups (e.g., ENSO phases) can inflate Type I error. Please add the results or clarify whether they have already been provided.

**Author response:**

We appreciate this suggestion to help improve the robustness of out result. We have conducted PERMDISP tests to verify PERMANOVA assumptions for all explanatory variables. The results show that our key findings are statistically valid: ENSO phases, SOI, and school all show equal multivariate dispersion (PERMDISP Pr > 0.1) while having significant PERMANOVA results (Pr < 0.001), confirming that these represent genuine centroid differences.

However, chlorophyll-a concentration and FADs showed both significant PERMDISP and PERMANOVA results, indicating potential dispersion artifacts rather than true centroid differences. All other variables showed non-significant PERMANOVA results. We have added all PERMDISP results to Supplementary File (S6 Table) and have updated our Methods and Results sections accordingly.

**Revised section:**

Lines 251 – 252: Before interpreting PERMANOVA results, Permutational Multivariate Analysis of Dispersion (PERMDISP) tests were verified using *vegan* package (version 2.6.4) [64] to test for equal multivariate dispersion among groups.

Lines 354 – 360: Beta diversity of the gut microbiome was significantly affected by school (PERMANOVA test: F _(14, 127)_ = 1.67, R^2^ = 0.16, Pr < 0.01,), ENSO (PERMANOVA test: F _(1, 140)_ = 3.03, R^2^ = 0.02, Pr < 0.01), SOI (PERMANOVA test: F _(1, 140)_ = 2.93, R^2^ = 0.02, Pr < 0.001), FADs (PERMANOVA test: F _(1, 140)_ = 2.77, R^2^ = 0.02, Pr < 0.01), and CHLA (PERMANOVA test: F _(1, 140)_ = 2.81, R^2^ = 0.02, Pr < 0.001) (Fig 3B). PERMDISP tests confirmed equal multivariate dispersion for the primary variables of ENSO, SOI and school, but not for FADs and CHLA (S6 Table).

3. Choice of distance & compositionality: You used unweighted UniFrac (presence/absence) after TSS normalization. Consider complementing with a compositional approach (Aitchison/CLR + Euclidean or PhILR) to ensure results are not an artifact of prevalence filtering or sequencing depth.

**Author response:**

We appreciate this careful thought. We have complemented our UniFrac analysis with a compositional approach using CLR transformation + Euclidean distance. Both analytical approaches show consistent results: ENSO remains significant (F _(1, 140)_ = 3.18, R^2^ = 0.02, Pr < 0.001), SOI effects are maintained (F _(1, 140)_ = 3.10, R^2^ = 0.02, Pr < 0.001). The concordance between phylogenetically-informed (UniFrac) and compositional (CLR) approaches demonstrates that our findings are robust across different distance metrics and are not artifacts of prevalence filtering or compositional constraints.

All these results can be found in supplementary file (S6 Table).

**Revised section:**

Lines 252 – 255: To verify robustness of results to distance metric choice, we also analyzed beta diversity using centered log-ratio (CLR) transformation followed by Euclidean distance, a compositional data analysis approach.

Lines 360 – 362: To ensure robustness across analytical approaches, we complemented unweighted UniFrac analysis with CLR transformation and Euclidean distance, which yielded consistent results (S6 Table).

4. Multiple testing and effect sizes in ANCOM-BC2: Please report the false discovery rate (FDR) adjustment and the effect sizes, which are bias-corrected log-fold changes with confidence intervals (CIs), for the five significant families.

**Author response:**

Thank you for this valuable suggestion to provide more comprehensive statistical reporting. We have expanded our ANCOM-BC2 reporting to include bias-corrected log-fold changes (effect sizes), 95% confidence intervals where calculable, and FDR-adjusted p-values (q-values) for all five significant bacterial families. Three families showed complete separation between ENSO phases (SE = 0), indicating highly discriminatory responses to environmental conditions. These detailed statistics are now provided in the Results section.

**Revised section:**

Lines 380 – 388: Five families showed significant abundance changes between ENSO events according to the ANCOM-BC2 differential abundance analysis (all p < 0.001 and q < 0.001). Fusobacteriaceae abundance significantly increased during the transition from La Niña to El Niño phase (LFC = 1.10, q < 0.001), while the abundances of Bacillaceae (LFC = 0.78, q < 0.001), Propionibacteriaceae (LFC = 0.76, q < 0.001), Beijerinckiaceae (LFC = 1.29, 95% CI: 0.51 to 2,06, q < 0.001), and Comamonadaceae (LFC = 1.52, 95% CI: 0.69 to 2.35, q < 0.001) significantly decreased during the transition phase (Fig 5A). Three families (Bacillaceae, Propionibacteriaceae, and Fusobacteriaceae) showed complete separation between ENSO phases (SE = 0).

Environmental data

Suggestions: Specify the exact products for SST/CHLA (e.g., MODIS/VIIRS product IDs), temporal windows used for extraction (8-day composites are listed), and any spatial averaging buffers around coordinates. Consider testing time-lagged windows (e.g., 2–8 weeks) to match biological integration times.

**Author response:**

We have added specific product details for environmental data extraction to our Methods section. Regarding time-lagged environmental effects, while testing SOI values at 1-8 week lags would be valuable for understanding biological integration times, the Bureau of Meteorology's daily SOI database only provides data from 2023 onwards, while our sampling occurred in December 2022 and March 2023. The historical data needed for lag analysis (November 2022 - February 2023) is not available in daily or weekly resolution from our primary SOI source. We have noted time-lagged analysis as an important future direction that could provide insights into delayed biological responses and better match biological integration times of gut microbiome to environmental changes.

**Revised section:**

Lines 125 – 130: Sea surface temperature and CHLA data were collected by National Aeronautics and Space Administration’s (NASA) Moderate Resolution Imaging Spectroradiometer (MODIS) sensor on Aqua satellite and obtained via NASA Earth Observations (NEO) platform (https://neo.gsfc.nasa.gov). Data were extracted as 8-day composites corresponding to sampling dates at exact sampling coordinates.

Statistics and modeling

Major points to address:

1. Alpha-diversity GAMs vs. non-normality: You note indices are non-normal but then fit Gaussian GAMs to continuous covariates. Either transform, fit appropriate distributions, or use non-parametric smoothers with robust inference; report diagnostics.

**Author response:**

Thank you for this suggestion to improve statistical consistency. We have revised our analysis to use generalized linear models (GAMs) with appropriate family distributions instead of Gaussian GAMs. Chao1 species richness and Shannon diversity now use Gamma family (appropriate for continuous positive data), while Simpson's evenness and relative abundance of core microbiome use quasi-binomial family (appropriate for bounded 0-1 data). School was included as a fixed effect to account for hierarchical structure.

We have updated Method and Result sections accordingly.

**Revised section:**

Lines 236 – 240: GAMs were fitted using appropriate family distributions based on the data characteristics of each diversity index: gamma family for Chao1 richness and Shannon diversity, and quasi-binomial family for Simpson's evenness index and relative abundance of core microbiome [64]. School was included as a random effect to account for hierarchical structure.

Lines 348 – 352: Chao1 richness and Simpson’s evenness showed significant non-linear relationships with SOI when analyzed using GAMs with appropriate family distributions (Chao1: Gamma family: R² = 0.004, t = -1.93, p = 0.05; Simpson’s evenness: quasi-binomial family: R² = 0.005, t = 2.33, p = 0.02) (Fig 3A). School effects were non-significant as random effects in both models.

2. SOI prediction with tiny n: The SOI models use five family-level predictors at the school level with n≈15—very small for random forests. Please conduct internal cross-validation that respects grouping (e.g., leave-one-school-out) and report those metrics. Current external validation is mixed (excellent for TGM; poor for GMB), consistent with overfitting and domain shift. Include permutation importance and partial-dependence plots.

**Author resonse:**

We have conducted leave-one-school-out internal cross-validation as suggested. The internal validation shows R² = 0.898 with RMSE = 5.2 and prediction standard deviation of 5.36. While continuous SOI prediction shows moderate precision, the model achieved perfect classification accuracy (100%) for distinguishing ENSO phases using the SOI threshold of 7 (S7 Table).

The mixed external validation performance (excellent for TGM, poor for GMB) results from domain shift rather than severe overfitting, given the strong internal validation performance and perfect ENSO phase classification. The GMB dataset's different SOI range (peak El Niño conditions absent from our training data) and storage conditions limit continuous prediction.

We have added permutation importance and partial dependence plots as requested in supplementary files (S2 and S3 Figs). Permutation importance analysis identified Propionibacteriaceae and Bacillaceae as the most important predictors of SOI. Partial dependence plots revealed that Fusobacteriaceae showed a negative relationship with predicted SOI, while the other four families exhibited positive relationships with SOI predictions. These patterns are fully consistent with our ANCOM-BC2 results, strengthening confidence in the biological meaningfulness of these microbiome-environment relationships.

We have updated related parts in Methods and Results sections, and added permutation importance and partial dependence plots as requested in the supplementary files.

**Revised section:**

Lines 281 – 282: To address potential overfitting of RFM because of the small sample size, we conducted leave-one-school-out internal cross-validation for the RFM.

Lines 404 – 406: While the internal cross-validation for RFM showed moderate precision (R² = 0.90, RMSE = 5.2), it achieved 100% accuracy in classifying ENSO phases using the SOI threshold of 7 (S7 Table).

Lines 409 – 414: Permutation importance analysis identified Propionibacteriaceae and Bacillaceae as the most important predictors of SOI in the random forest model (S2 Fig). Partial dependence plots revealed that Fusobacteriaceae showed a negative relationship with predicted SOI, while the other four families (Comamonadaceae, Beijerinckiaceae, Propionibacteriaceae, and Bacillaceae) exhibited positive relationships with SOI predictions (S3 Fig).

Ethics: The submission form says, “All necessary permits … were obtained,” but PLOS ONE requires details for field research/animal work (permit numbers, issuing authority, and capture, handling, and euthanasia methods or rationale). Please add these in the Methods and Ethics Statement.

**Author response:**

We have added a complete ethics statement to the Methods section. As confirmed by our co-authors at the Pacific Community, no field access permits were required for this study, as all samples originated from commercial fishery catches. The statement specifies that gut lining samples were collected from commercially caught skipjack tuna during routine operations, with no live animals handled for experimental purposes. All sampling followed standard commercial fishing practices and regional fisheries management protocols.

**Revised section:**

Lines 95 – 101: No field access permits were required for this study. All skipjack tuna (Katsuwonus pelamis) samples were obtained from commercial purse-seine fishery catches. Gut lining samples were collected during routine fisheries operations coordinated by the Pacific Community. No live animals were handled or subjected to experimental procedures as part of this research. All sampling procedures followed standard commercial fishing practices and regional fisheries management guidelines.

Results

Diet

Strengths include solid lab throughput, 16.3 million raw reads for diet analysis, 60 out of 75 samples containing non-Scombridae prey, and a dominance of fish species such as Myctophidae and Acanthuridae, with Cavoliniidae (pelagic gastropods) being particularly notable—this highlights the sensitivity of high-throughput sequencing (HTS). No prey family showed differences due to ENSO, which you interpret with caution.

Suggestions: Remind readers that read-based RA is semi-quantitative; you do use FOO and RA appropriately. Consider presenting Hill numbers and reporting sequencing depth summaries per sample alongside alpha metrics.

**Author response:**

We have added notes that read-based relative abundance should be interpreted as semi-quantitative due to inherent DNA amplification and sequencing biases.

Regarding Hill numbers, given that no significant dietary differences were detected across any grouping variables and our analysis already includes multiple complementary diversity metrics (Shannon, Chao1, Simpson's), we have opted to maintain our current analytical framework rather than add additional metrics that would not change our conclusions.

**Revised section:**

Lines 258 – 262: The frequency of occurrence (FOO) and relative abundance (RA) were calculated for each prey family using the microbiome package (version 1.23.1) [52] and visualized with a feeding strategy diagram [67]. Relative abundance based on read counts should be interpreted as semi-quantitative due to inherent biases in DNA amplification and sequencing efficiency across taxa.

Microbiome

Strengths: 2.9 M post-QC reads across 142 samples; significant effects of ENSO/SOI on richness/evenness and beta diversity; clear visualization; Vibrionaceae dominance supports freshness.

Major points to address:

1. Add PERMDISP results; include effect sizes (e.g., R² with CIs) and 95% CIs/ribbons on GAM plots; annotate group n’s on figures.

**Author response:**

We have added PERMDISP results confirming equal dispersion for key variables (S6 Table) and revised Figure 3 with detailed effect size reporting.

**Revised section:**

Lines 258 – 260: PERMDISP tests confirmed equal multivariate dispersion for the primary variables of ENSO, SOI and school, but not for CHLA (S6 Table).

2. Report ANCOM-BC2 statistics (adjusted p, LFC, CI) for the five families; indicate filtering thresholds and prevalence explicitly in the figure caption.

**Author response:**

We have expanded ANCOM-BC2 reporting to include log-fold changes, confidence intervals where calculable, and FDR-adjusted p-values (q-values), with filtering criteria specified in figure captions.

**Revised section:**

Lines 269 – 271: Prior to differential abundance testing, bacterial families were filtered to include only those with ≥10% prevalence across samples and ≥1% detection threshold.

Lines 380 – 388: Five families showed significant abundance changes between ENSO events according to the ANCOM-BC2 differential abundance analysis (all p < 0.001 and q < 0.001). Fusobacteriaceae abundance significantly increased during the transition from La Niña to El Niño phase (LFC = 1.10, q < 0.001), while the abundances of Bacillaceae (LFC = 0.78, q < 0.001), Propionibacteriaceae (LFC = 0.76, q < 0.001), Beijerinckiaceae (LFC = 1.29, 95% CI: 0.51 to 2,06, q < 0.001), and Comamonadaceae (LFC = 1.52, 95% CI: 0.69 to 2.35, q < 0.001) significantly decreased during the transition phase (Fig 5A). Three families (Bacillaceae, Propionibacteriaceae, and Fusobacteriaceae) showed complete separation between ENSO phases (SE = 0).

SOI prediction

Strength: Innovative use of microbiome to predict an environmental index; comparative modeling.

Concerns and requests:

1. Provide internal cross-validation that leaves out schools; with n≈15, report repeated CV variance.

**Author response:**

We conducted leave-one-school-out cross-validation with n=15 schools, reporting R² = 0.898, MSE = 27.26, RMSE = 5.2, and CV standard deviation of 5.36. Please see our response to Comment 2 in Statistics and modeling section.

2. Calibrate expectations in the text: training R²=0.991 and MSE=4.9 vs. poor performance on GMB suggests overfitting/domain mismatch; discuss implications for operational use.

**Author response:**

We have substantially revised the Discussion section to provide a more balanced assessment of the model's capabilities and limitations. We now present the model as demonstrating the biological principle that gut microbiome can detect ENSO phases, while being appropriately cautious about immediate operational use.

**Revised section:**

Lines 509 – 534:

The abundances of these five bacterial families demonstrated potential for environmental monitoring using a random forest model (RFM) but this must be interpreted cautiously given the small sample size. Importantly, internal cross-validation revealed that while SOI prediction showed moderate precision, the model achieved perfect accuracy in classifying ENSO phases. Same performance was observed in external validation using TGM dataset, indicating the potential of using RFM with fish gut microbiome abundances to monitor environmental change events.

However, both RFM and GAM showed low accuracy for the GMB test data, highlighting limitations in broader application of the model. The poor external validation performance could be related to two factors. First, SOI range mismatch: a large proportion of skipjack tuna samples in the GMB project were collected during peak El Niño phases (SOI: -20.2 to 9.7), whereas this study’s SOI range was -4.2 to 22.1. The lack of peak El Niño data in this study likely limited the model’s broader applicability, particularly its ability to predict SOI in El Niño phases. Second, preservation and storage effects: GMB gut samples had been stored for a long period with different preservation protocols compared to our freshly collected, immediately preserved samples. Microbiome composition can shift over time [75], potentially altering the bacterial family abundance patterns and affecting predictive accuracy.

This work represents proof-of-concept that gut microbiome composition and five bacterial families have the potential to distinguish ENSO phases in skipjack tuna. Future improvements to predictive models would benefit from systematic sampling across complete ENSO cycles to capture the full range of environmental conditions, particularly peak El Niño phases, larger sample sizes spanning multiple years, and standardized preservation protocols to ensure microbiome stability. Such comprehensive approaches would strengthen the operational deployment of microbiome-based environmental monitoring tools.

Discussion and Conclusions

Strengths: Balanced interpretation; limitations acknowledged (limited timeframe, storage/time-range discrepancies in validation sets).

Suggestions:

1. Expand discussion of spatial/temporal confounding and school effects; emphasize that two time points cannot separate ENSO from seasonal or spatial gradients.

**Author response:**

Thank you for this important point about the fundamental limitations of our study design. We have substantially expanded the Discussion section to address spatial/temporal confounding and school effects. The revised Discussion balances presenting our evidence for ENSO effects while being appropriately cautious about the limitations inherent in our sampling design.

**Revised section:**

Lines xxx – xxx: Spatial confounding might be less significant because the 4 - 5 degree latitudinal sampling range represents a modest variation relative to skipjack tuna migration patterns and regional stock distribution [40,41,85]. However, we acknowledge that our two-timepoint design cannot definitively separate ENSO effects from seasonal variations. Systematic sampling across multiple ENSO cycles at fixed locations would be required to conclusively separate ENSO effects from temporal and spatial variation.

2. Reframe functional inferences as hypotheses (immune vs. “probiotic” roles) given family-level resolution; suggest future metagenomics/metatranscriptomics.

**Author reponse:**

We have revised the Discussion to frame functional inferences about bacterial families as hypotheses rather than established facts.

**Revised section:**

Lines 489 – 495: These families have been associated with probiotic functions in metabolism, digestion, and nutritional provisioning in previous studies [90–95]. For example, species within Bacillaceae (e.g., *Bacillus subtilis*) are common probiotic additives in aquaculture feeds to improve fish digestion and immunity [90,93]. Genera within Beijerinckiaceae (e.g. *Bosea*) and Propionibacteriaceae (e.g. *Propionibacterium* and *Propioniciclava*) are closely related to high lipid and high protein feeding strategy in fish [91,92,94,96]

Lines 499 – 507: However, taxonomic identification at the family level has limitations for inferring function. Bacterial families are ecologically diverse, and functional capabilities can vary substantially among genera and species within families [99–101]. Therefore, the proposed associations between specific families and probiotic or immune-related functions should be considered hypotheses requiring validation rather than established mechanisms. Future studies employing metagenomics and metatranscriptomics would enable direct assessment of functional gene content and expression, providing insights into how these bacterial communities contribute to host metabolism, immunity, and stress responses during ENSO events.

Figures, Tables, and Presentation

Positives: The heatmap and NMDS are clear; the feeding-strategy plot is helpful.

Edits:

1. Add sample-size annotations, CIs on GAM smooths, and color-blind-safe palettes.

**Author response:**

We have added sample size annotations, 95% confidence intervals (shaded ribbons), and statistical results (t-values, p-values, R²) to Figure 3 as requested.

2. Minor language edits: “drifting or anchored FADs” (not “of anchored”), “Actinopterygii” (spelling), and “were sequenced” (verb agreement).

**Author response:**

We have corrected these language errors: fixed the spelling of "Actinopterygii," changed "drifting of anchored FADs" to "drifting or anchored FADs," and corrected the verb agreement for "sequenced" throughout the manuscript.

# Reviewer #2:

1. Scope and Limitations

Highlight more clearly in the Discussion and Conclusion that the study covers only 15 schools over 4 months. Emphasize the need for longer-term, multi-year sampling.

**Author response:**

We have expanded both the Discussion and Conclusion to explicitly highlight the study's temporal and spatial scope.

**Revised section:**

Lines 475 – 478: However, we acknowledge that our two-timepoint design cannot definitively separate ENSO effects from seasonal variations. Systematic sampling across multiple ENSO cycles at fixed locations would be required to conclusively separate ENSO effects from temporal and spatial variation.

Lines 527 – 534: This work represents proof-of-concept that gut microbiome composition and five bacterial families have the potential to distinguish ENSO phases in skipjack tuna. Future improvements to predictive models would benefit from systematic sampling across complete ENSO cycles to capture the full range of environmental conditions, particularly peak El Niño phases, larger sample sizes spanning multiple years, and standardized preservation protocols to ensure microbiome stability. Such comprehensive approaches would strengthen the operational deployment of microbiome-based environmental monitoring tools.

Lines 552 – 555: This proof-of-concept study demonstrates that skipjack tuna gut microbiome composition responds rapidly to ENSO transitions and can distinguish between climate phases. However, the findings are constrained by the short sampling period and small sample size, which cannot definitively separate ENSO effects from seasonal or spatial variation.

2. Diet Analysis

In the Results, acknowledge the low proportion of successful prey detection (40%).

In the Discussion, expand on causes (empty stomachs, degraded DNA, host DNA dominance) and suggest solutions (alternative markers, host DNA depletion).

In the Conclusion, note that diet results are preliminary compared to microbiome findings.

**Author response:**

We have expanded the Discussion to acknowledge the 40% prey detection rate and its causes, including empty stomachs, degraded DNA, and host DNA dominance. We now suggest practical solutions including alternative genetic markers and increased sequencing depth. In the Conclusion, we have explicitly noted that diet results should be considered preliminary compared to the more robust microbiome findings.

**Revised section:**

Lines 435 – 439: Although we employed host-blocking primers to reduce skipjack tuna DNA amplification, future studies could further improve detection rates through alternative genetic markers targeting different genes with varying degradation rates, increased sequencing depth to enhance detection of rare prey DNA, or multi-marker approaches to improve taxonomic coverage [77,78].

Lines 542 – 544: While skipjack tuna exhibited a diverse and opportunistic diet, diet results should be considered preliminary given the moderate prey detection rate.

3. Gut Microbiome

Add caution in the Discussion when linking bacterial families to immune or probiotic functions. Suggest future validation with metagenomics/metatranscriptomics.

**Author response:**

We have added cautionary language throughout the Discussion when discussing functional roles of bacterial families.

**Revised section:**

Lines 499 – 507: However, taxonomic identification at the family level has limitations for inferring function. Bacterial families are ecologically diverse, and functional capabilities can vary substantially among genera and species within families [99–101]. Therefore, the proposed associations between specific families and probiotic or immune-related functions should be considered hypotheses requiring validation rather than established mechanisms. Future studies employing metagenomics and metatranscriptomics would enable direct assessment of functional gene content and expression, providing insights into how these bacterial communities contribute to host metabolism, immunity, and stress responses during ENSO events.

4. Modeling

Expand in the Results/Discussion on why the Random Forest model failed with the GMB dataset (dataset differences, SOI range, storage issues).

**Author response:**

We have expanded the Discussion to provide a more detailed explanation of why the Random Forest model performed poorly on the GMB dataset.

**Revised section:**

Lines 516 – 526: However, both RFM and GAM showed low accuracy for the GMB test data, highlighting limitations in broader application of the model. The poor external validation performance could be related to two factors. First, SOI range mismatch: a large proportion of skipjack tuna samples in the GMB project were collected during peak El Niño phases (SOI: -20.2 to 9.7), whereas this study’s SOI range was -4.2 to 22.1. The lack of peak El Niño data in this study likely limited the model’s broader applicability, particularly its ability to predict SOI in El Niño phases. Second, preservation and storage effects: GMB gut samples had been stored for a long period with different preservation protocols compared to our freshly collected, immediately preserved samples. Microbiome composition can shift over time [75], potentially altering the bacterial family abundance patterns and affecting predictive accuracy.

Suggest adding environmental covariates and full ENSO cycle sampling for stronger predictive models.

**Author response:**

Regarding environmental covariates, we note that our analysis already incorporated multiple environmental variables including sea surface temperature (SST), chlorophyll-a concentration (CHLA), and SOI in our statistical models (as shown in Table 3 and reported in the Results). The random forest model specifically focused on bacterial family abundances as predictors to assess the microbiome's intrinsic predictive capability.

We have added emphasis on the critical need for full ENSO cycle sampling in the Discussion.

**Revised section:**

Lines 530 – 534: Future improvements to predictive models would benefit from systematic sampling across complete ENSO cycles to capture the full range of environmental conditions, particularly peak El Niño phases, larger sample sizes spanning multiple years, and standardized preservation protocols to ensure microbiome stability.

5. Figures

Expand figure legends for clarity.

Add a workflow diagram (Methods) and a conceptual diagram (Discussion/Conclusion).

**Author response:**

We have expanded figure legends throughout the manuscript for clarity, particularly for Figures 3 and 5, adding detailed statistical information and filtering criteria.

Regarding workflow and conceptual diagrams, we believe the detailed Methods section and structured Results/Discussion adequately convey the study design and conceptual framework.

6. Methods & Ethics

Move detailed PCR conditions to Supplementary Material.

Add permit numbers in the Ethics Statement.

**Author response:**

Regarding PCR conditions, we have opted to retain these details in the main Methods section as they are essential for reproducibility and represent standard practice in molecular ecology publications. The conditions are clearly organized under separate subheadings for diet and microbiome analyses, making them easy to locate for readers attempting to replicate our methods.

We have added a complete ethics statement to the Methods section. As confirmed by our co-authors at the Pacific Community, no field access permits were required for this study, as all samples originated from commercial fishery catches. The statement specifies that gut lining samples were collected from commercially caught skipjack tuna during routine operations, with no live animals handled for experimental purposes. All sampling followed standard commercial fishing practices and regional fisheries management protocols.

**Revised section:**

Lines 95 – 101: No field access permits were required for this study. All skipjack tuna (*Katsuwonus pelamis*) samples were obtained from commercial purse-seine fishery catches. Gut lining samples were collected during routine fisheries operations coordinated by the Pacific Community. No live animals were handled or subjected to experimental procedures as part of this research. All sampling procedures followed standard commercial fishing practices and regional fisheries management guidelines.

7. Language

Avoid repeating statistics in both Results and Discussion.

Correct minor grammar (e.g., “in study” → “in this study”) and standardize references.

**Author response:**

We have reviewed both Results and Discussion sections to eliminate redundant statistical reporting. Statistics are now reported primarily in the Results section, with the Discussion focusing on biological interpretation and significance without repeating exact statistical values. We have also corrected minor grammatical issues including "in study" to "in this study" and standardized reference formatting throughout the manuscript.
