# Supplementary material for: Diet and gut microbiome of skipjack tuna (Katsuwonus pelamis) as indicators of environmental changes
Source: PLoS One. 2026 Apr 27;21(4):e0346882. doi: 10.1371/journal.pone.0346882 (PMC13119836; doi:10.1371/journal.pone.0346882)
Supplement: Attachment — Submitted filename: rebuttal_letter_revision2.docx [file pone.0346882.s014.docx]

# Reviewers' comments:

### Reviewer #1:

I thank the authors for their detailed and thoughtful responses to the initial review and for the substantial revisions made to the manuscript. Overall, the revised version shows a clear improvement in statistical rigour, transparency, and balanced interpretation. Many of the methodological and interpretative concerns raised in the first round have been carefully addressed, and I appreciate the effort invested in strengthening the analyses and presentation.

The authors deserve special recognition for their efforts:

(i) adding PERMDISP tests to support PERMANOVA interpretations;

(ii) incorporating a compositional (CLR-based) beta-diversity analysis alongside UniFrac;

(iii) revising alpha-diversity analyses to account for sequencing depth;

(iv) expanding ANCOM-BC2 reporting to include effect sizes and multiple-testing correction; and

(v) substantially improving the Discussion by acknowledging design limitations and reframing functional inferences more cautiously.

Most of my original comments have therefore been satisfactorily addressed. I have only a small number of remaining points, primarily related to clarity and consistency of interpretation rather than additional analyses.

1. Interpretation of microbiome vs. diet responses

The manuscript now appropriately emphasises the short, two-time-point sampling design and its limitations. However, in a few places, the text still implies that the absence of detectable diet changes, combined with microbiome shifts, provides evidence that gut microbiomes respond more rapidly or directly to ENSO forcing. While this interpretation is plausible, it cannot be demonstrated with the current design and remains inferential.

I recommend consistently framing this contrast as a hypothesis or inference, rather than as supporting evidence, throughout the Discussion. This would further align the interpretation with the acknowledged limitations of temporal and spatial confounding.

**Author response:**

We thank the reviewer for this important clarification. We have revised the Discussion and Conclusions to consistently frame the microbiome-diet contrast as an inference rather than as supporting evidence.

**Revised section:**

Lines 460 – 461: In contrast to diet, the gut microbiome of skipjack tuna showed detectable response to environmental changes during the study period.

Lines 466- 467: This study may be the first to document detectable ENSO-associated changes in a marine fish gut microbiome over a four-month transitional period.

Lines 547 – 550: No significant relationship was found between diet composition and ENSO events potentially due to the high empty stomach rate, limited sample size, and the short sampling timeframe.

The gut microbiome showed detectable changes associated with ENSO events and SOI level, with significant changes in species richness and evenness.

2. Treatment of school effects across analyses

The rationale for aggregating microbiome data at the school level for SOI modelling is statistically sound, and I agree that SOI is inherently a school-level variable. However, the manuscript alternates between describing school as a fixed effect and as a random effect in different parts of the Methods and Results.

For clarity, I suggest briefly harmonising this language and explicitly stating how school effects are handled in each analytical context (PERMANOVA, GAMs, and predictive models). This will help readers follow the hierarchical structure of the analyses more easily.

**Author response:**

We thank the reviewer for this helpful suggestion. We have revised the Methods section to explicitly state how school is handled in each analysis type.

For PERMANOVA analysis, we added clarification that school is treated as a categorical fixed effect. For GAMs and predictive models, there are already school-handling methods, which we have retained.

**Revised section**:

Lines 251 – 253: Before interpreting PERMANOVA results, Permutational Multivariate Analysis of Dispersion (PERMDISP) tests were verified using *vegan* package (version 2.6.4) [64] to test for equal multivariate dispersion among groups, treating school as a categorical fixed effect.

3. Predictive modeling and strength of claims

The additional leave-one-school-out cross-validation, permutation importance, and partial-dependence plots substantially strengthen the modelling section. The revised Discussion is also more balanced in acknowledging mixed external validation and domain-shift effects.

That said, given the minimal sample size (n ≈ 15 schools), repeated emphasis on “100% ENSO phase classification accuracy” risks being interpreted as stronger evidence of predictive power than is warranted. I recommend consistently qualifying these results as exploratory and proof-of-concept, rather than predictive in an operational sense, whenever they are mentioned.

**Author response:**

We thank the reviewer for this important suggestion to ensure appropriate interpretation of our predictive modeling results. We have revised the manuscript to consistently qualify these results as exploratory and proof-of-concept throughout.

**Revised section:**

Lines 405 – 407: While the internal cross-validation for RFM showed moderate precision (R² = 0.90, RMSE = 5.2), it achieved perfect ENSO phase classification in this proof-of-concept analysis (100% accuracy, n = 15 schools) using the SOI threshold of 7 (S7 Table).

Lines 516 – 517: In this proof-of-concept study, internal cross-validation revealed that while SOI prediction showed moderate precision, the model achieved perfect accuracy in classifying ENSO phases. Similar classification performance was observed in external validation using TGM dataset, suggesting that this exploratory approach has the potential for further investigation with larger sample size across complete ENSO cycles.

Lines 537 – 538: Such comprehensive approaches would strengthen further operational deployment of microbiome-based environmental monitoring tools.

4. Effect sizes and variance explained

Many microbiome–environment relationships explain a relatively small proportion of total variance (e.g., R² ≈ 0.02). This is not problematic and is common in ecological microbiome studies, but explicitly acknowledging this in the text would further strengthen the manuscript’s interpretive balance and prevent over-interpretation of statistically significant effects.

**Author response:**

We thank the reviewer for this suggestion. We have added a clarifying statement to the Discussion section that contextualizes the R² values.

**Revised section:**

Lines 470 – 473: While ENSO and SOI explained a relatively small proportion of total variance in microbiome composition, such modest effect sizes are typical in ecological microbiome studies and can still reflect ecologically important responses to environmental change.

## Reviewer #2:

The revisions have resolved all major concerns from the previous round, including statistical robustness, limitation acknowledgments, and balanced interpretations.

**Author response:**

We thank Reviewer #2 for their positive assessment and for confirming that our revisions have adequately addressed the concerns raised in the previous round. We appreciate their constructive feedback throughout the review process, which has substantially strengthened the manuscript.
